# Panicle-Cloud: An Open and AI-Powered Cloud Computing Platform for Quantifying Rice Panicles from Drone-Collected Imagery to Enable the Classification of Yield Production in Rice

**DOI:** 10.34133/plantphenomics.0105

**Published:** 2023-10-16

**Authors:** Zixuan Teng, Jiawei Chen, Jian Wang, Shuixiu Wu, Riqing Chen, Yaohai Lin, Liyan Shen, Robert Jackson, Ji Zhou, Changcai Yang

**Affiliations:** ^1^ Digital Fujian Research Institute of Big Data for Agriculture and Forestry, College of Computer and Information Sciences, Fujian Agriculture and Forestry University, Fuzhou 350002, China.; ^2^ State Key Laboratory of Crop Genetics & Germplasm Enhancement, academy for Advanced Interdisciplinary Studies, Nanjing Agricultural University, Nanjing 210095, China.; ^3^ Ningxia Academy of Agriculture and Forestry Sciences, Yinchuan 750002, China.; ^4^ College of Mechanical and Electrical Engineering, Fujian Agriculture and Forestry University, Fuzhou 350002, China.; ^5^ Cambridge Crop Research, National Institute of Agricultural Botany (NIAB), Cambridge CB3 0LE, UK.; ^6^ Key Laboratory of Smart Agriculture and Forestry (Fujian Agriculture and Forestry University), Fujian Province University, Fuzhou 350002, China.; ^7^ Center for Agroforestry Mega Data Science, School of Future Technology, Fujian Agriculture and Forestry University, Fuzhou 350002, China.

## Abstract

Rice (*Oryza sativa*) is an essential stable food for many rice consumption nations in the world and, thus, the importance to improve its yield production under global climate changes. To evaluate different rice varieties’ yield performance, key yield-related traits such as panicle number per unit area (PNpM^2^) are key indicators, which have attracted much attention by many plant research groups. Nevertheless, it is still challenging to conduct large-scale screening of rice panicles to quantify the PNpM^2^ trait due to complex field conditions, a large variation of rice cultivars, and their panicle morphological features. Here, we present Panicle-Cloud, an open and artificial intelligence (AI)-powered cloud computing platform that is capable of quantifying rice panicles from drone-collected imagery. To facilitate the development of AI-powered detection models, we first established an open diverse rice panicle detection dataset that was annotated by a group of rice specialists; then, we integrated several state-of-the-art deep learning models (including a preferred model called Panicle-AI) into the Panicle-Cloud platform, so that nonexpert users could select a pretrained model to detect rice panicles from their own aerial images. We trialed the AI models with images collected at different attitudes and growth stages, through which the right timing and preferred image resolutions for phenotyping rice panicles in the field were identified. Then, we applied the platform in a 2-season rice breeding trial to valid its biological relevance and classified yield production using the platform-derived PNpM^2^ trait from hundreds of rice varieties. Through correlation analysis between computational analysis and manual scoring, we found that the platform could quantify the PNpM^2^ trait reliably, based on which yield production was classified with high accuracy. Hence, we trust that our work demonstrates a valuable advance in phenotyping the PNpM^2^ trait in rice, which provides a useful toolkit to enable rice breeders to screen and select desired rice varieties under field conditions.

## Introduction

Rice (*Oryza sativa*) is one of the most important crops in the world [1]. Many rice-consuming nations rely on the crop as their staple food, indicating the significance of maintaining its production globally [2]. Nevertheless, a changing climate is putting global rice production at risk [3], including increasing heat and drought stresses that led to fluctuating rice yield production in China during the past decade [4]. This resulted in the change of rice breeding strategies toward more climate-resilient and sustainable crop production system in recent years [5].

To improve crop yields, good indicators for selecting more adaptive rice plants with better yield performance are key yield components such as panicle number per unit area (PNpM^2^), spikelet number per panicle (SNpP), as well as grain-related traits including grain number per panicle (GNpP), grain length and width, and thousand grain weight (TGW) [6]. These agronomic traits were typically scored by specialists following traditional sampling methods, either under field conditions or during postharvest handling, which were also key to the assessment of genetic gain, yield formation, and yield production [7,8]. In comparison with SNpP, GNpP, or other grain-level traits that were difficult to measure in the field, PNpM^2^ is relatively easy to phenotype, when rice panicles partially or fully emerged and their morphological and color features became distinct at the rice canopy [7]. In fact, to quantify panicle-like objects, many computer vision and artificial intelligence (AI)-powered methods have been developed [9]. For example, deep learning (DL) models such as You Only Look Once (YOLO) have been used to score rice panicles from rice images [10]; color and texture features of rice panicles were used to detect panicles [11]; pixel-grouping and superpixel-based segmentation were used by the Panicle-SEG to detect panicles [12]; DL techniques (e.g., deep fully convolutional neural network) were integrated into the PanicleNet to segment rice panicles [13].

It is noticeable that many published studies were applied to a relatively small scale of indoor or in-field experiments and with limited accessibility due to the use of proprietary hardware and software or the lack of publicly available training datasets, making these solutions difficult to be utilized by the broader plant research community. More importantly, there are almost 40,000 types of rice in the world, many of which possess very different panicle morphologies and developmental profiles, from heading to grain filling [14]. Hence, it is still challenging to perform in-field phenotypic analysis of rice panicles at a large scale and at the right time [2,15]. In fact, many breeders, agronomists, and crop researchers were still manually scoring key yield-related traits in field trials, which was time-consuming, laborious, and prone to error [16]. Because of the rapidly changing climates, breeders and plant researchers needed to accelerate the process of crop selection, requiring the examination of thousands of crop varieties at multiple sites and across several seasons. This required a relatively short processing cycle due to a small window of time to decide the strategy of field experiments [17]. As a result, more powerful data collection and reliable analytic toolkits are needed in crop improvement.

To collect large-scale rice imagery under field conditions, current methods range from satellite remote sensing, light airplane, to unmanned aerial vehicles (UAVs; such as drones) and ground-based phenotyping devices. These phenotyping devices had diverse advantages and disadvantages: (a) Satellite sensing system was long distance and ultrascale, but its imagery was relatively low resolution, making it difficult to capture small organ-level objects such as rice panicles [18]; (b) light airplanes were normally equipped with hyper- and multispectral image sensors for large-scale field surveillance; still, it was difficult to acquire clear panicle-level objects due to high flight altitudes and speed [19]; (c) low-altitude UAVs fitted with high-resolution red–green–blue (RGB) cameras have been used in field phenotyping popularly, which were reported for their capabilities to acquire imagery with organ-level resolutions when flying at low altitudes [20]; (d) ground-based devices were usually used to collect very high-resolution 2-dimensional (2D) or 3D plant images at fixed locations and angles; however, their applications were restricted in scalability because of their mobility in rice paddy fields [21]. Among the above phenotyping approaches, drone-based phenotyping clearly possesses advantages in flexibility, scalability, and cost effectiveness, which is likely to be useful when collecting rice panicle signals at a large scale.

Besides data collection, varied computational analysis techniques have been developed to quantify panicle-like objects, including pixel-based grouping and object-based AI [i.e., machine learning (ML) and DL techniques] detection. ML and DL detection demonstrated unique values in measuring small objects because information such as object positions, pixel connectivity, and other low-level features (e.g., shapes, edges, and textures) could be learned by AI models, differentiating panicle-like objects from their surrounding pixels [10,11]. A range of learning architectures has been trialed in this field, including Mask R-CNN [22], YOLOv4 [23], YOLOv5 [10], MobileNetv2 [23], R-FCN [24], and FPN-Mask [25]. For example, the Panicle-Mask algorithm based on Mask R-CNN was established to quantify panicle regions [22]; an improved YOLOv4 model was developed to detect curved panicles [23]; an improved fully convolutional network was developed to detect rice panicles from drone-collected images [24]; an FPN-Mask model was built to identify panicles and assess yield performance based on the leaf-to-panicle ratio of specialists’ yield assessment [25]; recently, a DL-based pipeline was proposed to detect rice panicles under different nitrogen treatments from time-series images using a combination of YOLO v5, ResNet50, and DeepSORT models [26].

The above methods have made valuable progresses in detecting rice panicles. Still, the generality and scalability of these methods can be improved. Both low-level (e.g., morphological and color space) and high-level (e.g., semantic information such as objects and scenes) features should be incorporated into the recognition of panicle-like objects [11,27]. In particular, because different rice varieties possess dissimilar panicle morphologies (e.g., shape, color, size, and spatial positions) and developmental paces during the reproductive phase (e.g., from heading to grain filling), AI models need to incorporate both developmental (i.e., the growth stage to phenotype rice panicles) and morphological features (i.e., the level of image resolution and organ signals sufficient for object detection), through which suitable learning models could be selected to provide reproducible phenotypic analysis of rice panicles from hundreds of varieties.

Finally, it is widely recognized that the bottleneck of applying AI techniques to plant research is high-quality training datasets, making ML/DL models difficult to be trained and verified effectively [10,23]. Unlike the openly available Global Wheat Head Detection (GWHD) dataset [28] where a large and diverse dataset of labeled RGB images of wheat spikes has been made available to public, an open rice panicle detection dataset still does not exist for researchers to develop and benchmark their rice panicle detection models. Furthermore, much research still has not made training data and detailed method implementation fully available [29], which is key to facilitate plant researchers and breeders to accelerate ongoing research to catch up with the pace of global climate changes.

Here, we present Panicle-Cloud, an open and AI-powered cloud computing platform that is capable of quantifying rice panicles from large-scale drone-collected rice imagery. To train AI-powered panicle detection models, we first created an open diverse rice panicle detection (DRPD) dataset, which was annotated by a group of rice specialists. Then, to facilitate nonexperts to use and compare analysis results powered by AI modeling, we integrated several DL models into the Panicle-Cloud platform, so that users could select a suitable model to quantify panicles. To verify the right timing and image resolution to phenotype rice panicles, we performed drone phenotyping at 3 flight attitudes and at 4 key growth stages (i.e., heading, flowering, early grain filling, and middle grain filling) under field conditions. To validate the model-assisted panicle detection of the PNpM^2^ trait, we carried out correlation analysis. Through an iterative model improvement approach, we developed a preferred DL model called Panicle-AI, which helped us yield the best panicle detection accuracy. Finally, the Panicle-Cloud platform was applied to a 2-season rice breeding trial, and a supervised ML model was established to classify yield performance from hundreds of rice varieties using the PNpM^2^ trait. We found that the PNpM^2^ trait produced by the platform could classify rice yield production with high accuracy, which enabled rice breeders to screen and select preferred rice varieties.

## Materials and Methods

### Two-season field experiments

**Fig. 1.**
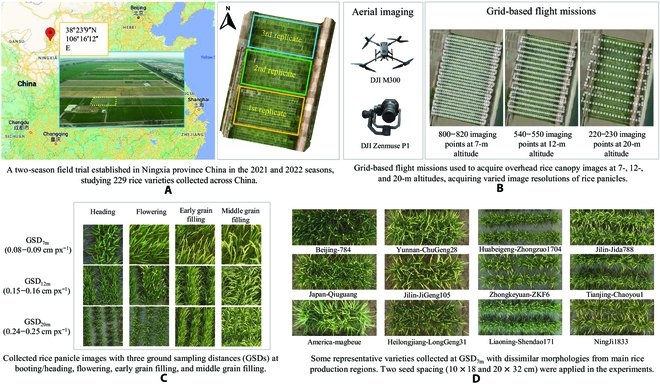
The 2-season field trial and rice panicle images acquired by drones. (A) Geo-location of the field trial site. (B) Three grid-based flight missions designed to image rice panicles using drones. (C) Representative rice panicle imagery acquired at 3 different GSDs and 4 key growth stages. (D) Representative rice varieties examined by drone-based aerial phenotyping at 7-m altitude.

In this study, we selected 229 rice varieties in a 2-season field experiment at the Crop Research Institute, Ningxia Academy of Agriculture and Forestry, Ningxia China (38°23′9″ N, 106°16′12″ E; Fig. 1A). For the 2021/2022 rice growing seasons, these varieties were sowed in plots with 10 columns and 2 rows per plot, 20 plants in total. The spacing between each plot was 25 cm. Each variety had 3 or more replications, with a total of 1,374 plots studied per season. To test the accuracy of our DL models and facilitate preferred rice breeding practices, we introduced 2 planting densities (A: 10 × 18 cm and B: 20 × 32 cm) in the field experiments.

### Manual scoring and ground truthing

To validate DL models developed in our study, technicians manually scored rice panicles from the drone-collected images together with breeders. To ensure the reliability of the manual scores, 3 technicians were arranged to label the rice panicles per plot from randomly selected collected images using the LabelImg software [30], whereas field workers counted the PNpM^2^ trait based on the number of rice panicles in a given plot. Results from the 2 approaches were cross-referenced and recorded as manual scoring.

In addition, to facilitate the yield classification in the 2-season trial, we produced plot-based rice grain production (in kilograms per plot): (a) We weighed rice grains harvested from the field experiments; (b) rice spikelets were left to dry in an open environment for 2 days, followed by threshing using a seed thresher; (c) we weighed the dry rice grains with an electronic scale and obtained grain production (in kilograms per plot).

### Drone-based phenotyping of rice panicles

Because we needed to cover the 1,374 plots within a relatively short period of time due to different rates of grain filling of 229 rice varieties, we used a high-end drone (DJI M300, DJI Technology, Shenzhen China) equipped with a DJI Zenmuse P1 camera (35-mm focal length lens, with a maximum image resolution of 8,192 × 5,460 pixels) to carry out aerial phenotyping (Fig. 1B, left). Three flight missions were designed using the single grid-based mission mode (Fig. 1B, right). Images were stored in JPG format using the standard RGB color standard.

To identify the suitable altitude for imaging rice panicles, we flew the drone at 3 altitudes (Fig. 1C), i.e., 7 m (with a ground sampling distance, GSD_7m_, which equals to 0.08 to 0.09 cm·pixel^−1^), 12 m (GSD_12m_ = 0.15 to 0.16 cm·pixel^−1^), and 20 m (GSD_20m_ = 0.24 to 0.25 cm·pixel^−1^). When conducting flight missions, aerial imaging was performed at 4 key growth stages when most of the varieties entered a specific growth stage (i.e., heading, flowering, early grain filling, and middle grain filling), between July and August in the 2 seasons. Many series of raw aerial images (1,200 GB) were collected from the field experiments.

### Selected rice varieties for the experiments

To improve the generalization and usability of our AI-powered solutions, the 229 rice varieties selected were cultivated in many regions such as China, America, and Japan (e.g., Beijing784, America-magbeue, and Japan-Qiuguang), covering many panicle morphological features (Fig. 1D). More importantly, these varieties’ yield performance and panicle morphological features were known to be different [31], which were suitable for building a predictive model for yield-based classification.

### The analysis workflow for establishing the Panicle-Cloud platform

**Fig. 2.**
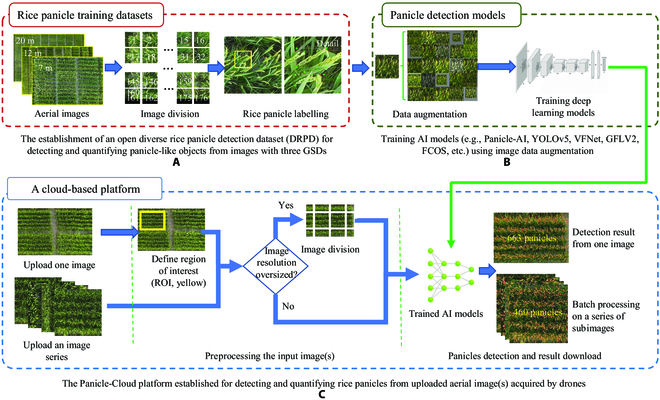
The analysis workflow of the Panicle-Cloud platform for detecting rice panicles from drone-collected imagery. (A) Image preprocessing (numbers representing the number of divided subimages) and rice panicle labeling. (B) Image augmentation and training AI models. (C) The establishment of the Panicle-Cloud platform for panicle detection and counting.

As the Panicle-Cloud platform is the center for out AI-powered solution, we followed a 3-step workflow to establish the cloud computing platform (Fig. 2), including (a) building an open DRPD dataset, which consisted of annotated aerial rice panicles imaged at 3 different altitudes, with 512 × 512-pixel image resolution and 5,372 annotated subimages (cropped from raw drone-collected images) in total (Fig. 2A); (b) training DL models with image augmentation functions [32] to increase the training datasets and reduce overfitting, including Panicle-AI (our tailored DL model), YOLOv5 [33], VFNet [34], FCOS [35], GFLv2 [36], and RetinaNet [37] (Fig. 2B); and (c) establishing the Panicle-Cloud platform for rice panicle counting, which detected and quantified panicle-like objects using integrated AI models from an uploaded image or image series with automatic image division (Fig. 2C).

### An open DRPD dataset

As training datasets are essential for building reliable DL models, we therefore developed the open DRPD dataset, which contained 5,372 RGB subimages cropped from raw aerial imagery taken at 3 different altitudes, i.e., GSD_7m_, GSD_12m_, and GSD_20m_. In total, DRPD included 259,498 labeled rice panicles with varied morphological features collected at 4 key growth stages, i.e., heading (1,903 subimages), flowering (1,676 subimages), early grain filling (1,235 subimages), and middle grain filling (558 subimages). The DRPD dataset consisted of (a) images acquired at 7-m altitude (i.e., GSD_7m_), including 106,878 labeled rice panicles and 3,810 rice subimages (27 to 30 panicles per subimage); (b) at 12-m altitude (i.e., GSD_12m_), including 71,404 labeled rice panicles and 1,004 subimages (65 to 70 panicles per subimage); and (c) at 20-m altitude (i.e., GSD_20m_), including 558 subimages and 81,216 labeled rice panicles (140 to 150 panicles per subimage). As our main focus was to perform high-accuracy panicle-like object detection, the 7-m subimages therefore accounted for just over 70% of the DRPD dataset. To assess the suitable growth stage for panicle phenotyping, 180 plot-level images were cropped from the acquired aerial images, and 760 subimages were used in total.

### The optimized learning architecture of Panicle-AI

**Fig. 3.**
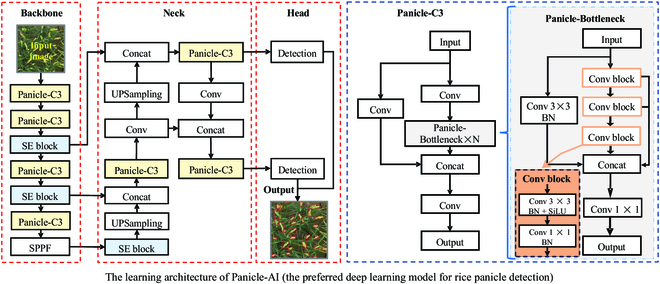
The proposed Panicle-AI model and its architecture, which was used as a preferred AI model for detecting rice panicles.

Among the DL models integrated in the Panicle-Cloud platform, the preferred model, Panicle-AI, was developed using the YOLOv5 baseline architecture. Because the successive C3 blocks in the learning architecture could lead to losses of features for small objects [38], we therefore created a Panicle-Bottleneck (PB) block in each C3 block. The PB block had 2 branches (Fig. 3, right): (a) a 3 × 3 convolution with a batch normalization (BN) and (b) 3 stacked convolution blocks, which consisted of a 3 × 3 convolution, a BN, and sigmoid linear units, as well as a 1 × 1 convolution with a BN. The output of the previous convolution block fed into the next convolution block, which accepted the previous feature and then further processed it in a larger receptive field.

In the PB, the first branch feature map and 3 convolution block feature maps were used to concatenate and generate rich context semantic information, which was key to capture high-level vision-based rice panicle features with noisy field backgrounds. This was followed by a 1 × 1 convolution, which recovered the number of channels of the output feature map. The tailored PB helped us obtain detailed features of rice panicles, which improved the detection accuracy of Panicle-AI greatly compared with a standard YOLOv5 architecture. In addition, because rice panicles were often partially occluded by leaves or adjacent panicles, we therefore introduced the squeeze-and-excitation (SE) block [39] to enhance the channel attention module to represent features of occluded objects. By using the SE block twice in the backbone and once in the neck (light-blue-colored rectangles; Fig. 3, left), we enhanced the selectivity of strongly semantic information together with location information of low-level features, improving the feature extraction ability of Panicle-AI.

As the binary cross-entropy loss function was not suitable for training dense rice panicles due to imbalance foreground and background classes, the locations between prediction frames, and the loss calculations, we therefore calculated the classification loss and confidence loss in the Panicle-AI model using the VariFocal loss function [34], which was used to train a dense panicle detector to predict the confidence and localization accuracy of intersection over union aware classification scores. Finally, as most of the rice panicles were relatively small in collected aerial images, we only used 2 layers of detection heads in the model.

### Rice yield classification model

We chose 3 factors to classify yield production from hundreds of rice varieties trialed, including the PNpM^2^, SNpP, and TGW (in kilograms). The PNpM^2^ trait was derived from the preferred Panicle-AI model integrated into the Panicle-Cloud platform, whereas the SNpP and TGW traits were sampled by field workers together with historic datasets retrieved from the National Rice Data Center, China (www.ricedata.com). When building the yield classification model, PNpM^2^, SNpP, and TGW were input parameters, and yield levels were the targets; 4 supervised ML models (i.e., decision trees [40], CatBoost [41], LightGBM [42], and random forest [43]) were used, followed by the *k*-fold cross-validation [44] to select a model with the best prediction accuracy. To support yield classification, a total of 480 plots (80 varieties, 3 replicates, and 2 seasons), their PNpM^2^ results, and plot yields were selected according to their historic yield records [45]. These datasets were then randomly divided into 70% (for training and validation) and 30% (for testing) sets in the yield modeling (Table S1).

### Training strategy and evaluation metrics

When training DL models integrated into the Panicle-Cloud platform, we used 2,286 subimages from the DRPD dataset for training (60%), 1,143 for testing (30%), and 381 for validation (10%) at the 7-m altitude; 602 images for training (60%), 302 for testing (30%), and 100 for validation (10%) at the 12-m altitude; and 344 images for training (60%), 168 for testing (30%), and 56 for validation (10%) at the 20-m altitude. DL models were trained on workstations using resized subimages (640 × 640 pixels). The workstation was running on Ubantu 18.04 operating system, equipped with an NVIDIA GeForce RTX 3090 (24-GB memory) and the PyTorch framework [46]. During model training, the batch size was set to 4, with an initial value of 0.01 and the decay of 0.0005.

In addition, during the model training, image augmentation techniques such as mosaic, cut mix, basic scaling, cropping, and rotation (Fig. 2B) were applied to mimic in-field imaging conditions, enrich training datasets through image transform operations, and expand the labeled data in the DRPD to optimize the performance of trained AI models. When training DL models, we often adjusted DL models’ hyperparameters to achieve a better detection result. Evaluation metrics and loss functions such as Precision, recall, F1-score, and the mean average precision over 0.5 intersection over union threshold (mAP@.5) were used to evaluate the model performance during training (see Fig. S1 for training the Panicle-AI model using the 7-m aerial imagery, i.e., subimages with GSD_7m_).

We used the coefficient of determination (*R*^2^) and root mean square error (RMSE) to evaluate computational results against manual scoring For yield classification, we selected some representative rice varieties (e.g., Ningxia Fuyuan 4, and Ninggeng 50) as yield-benchmark varieties [47]. Rice varieties were classified into 3 yield classes after comparing with the benchmarks. Varieties with yields of ≤90% of the benchmark yield were classified as low-yielding, those >90% and <110% of the benchmark yield were classified as medium yielding, whereas those ≥110% of the benchmark yield were classified as high yielding. We compared the 3 yield categories predicted by the ML model with those obtained through postharvest handling, so that the performance of the yield classification model could be evaluated with ground truthing.

### A cloud computing platform and software implementation

The Panicle-Cloud platform was developed using the Django framework [48], which was suitable for different web architectures. Using the Django toolkits such as object-relational mapping, form processing, authentication systems, and caching systems, we could quickly build front-end and back-end web applications for the Panicle-Cloud platform. The platform applied the browser–server structure and the model–view–controller (MVC) architecture, which included functions from uploading images, loading AI models, and performing AI-powered trait analysis and results display. The system architecture, key functions, and database management components were designed according to the MVC design. When implementing the cloud system, the back-end system was developed Python language with JQuery JavaScript library as the front-end development toolkit. MySQL database management system was used to manage cloud-based datasets, including image metadata, variety ID, user information, and analysis results.

We also included the DRPD dataset in the platform, so that other developers could utilize it for their own vision-based or AI development. Besides the DL models, the platform also included open scientific libraries such as OpenCV [49], MMDetection [50], and Python Imaging Library [51], which were used to support image cropping and resizing, image calibration, and image processing on the cloud platform. The development environment of the Panicle-Cloud platform was AliCloud (a subsidiary of Alibaba Group, Singapore), through which the Panicle-Cloud platform was deployed (http://ai-panicle.com:32123) and is accessible for academic use. In addition, detailed runtime environment and configuration are detailed in our GitHub repository (see Data Availability).

## Results

### The preferred drone-based panicle phenotyping approach

**Fig. 4.**
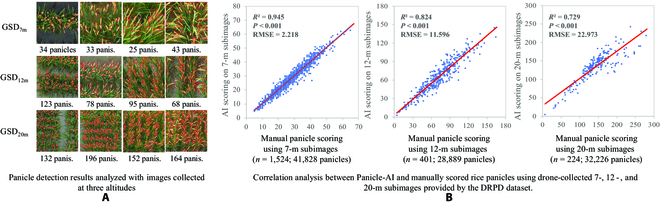
(A) Panicle detection and counting results on rice images acquired at 7-, 12-, and 20-m altitudes, across 4 key growth stages. (B) Correlation analysis performed between manually counted results and Panicle-AI-derived results.

First, to identify an ideal flight altitude and the right timing for phenotyping rice panicles, we used the Panicle-AI model to detect panicles from subimages with the 3 GSDs and thus the PNpM^2^ trait computed for all the varieties (4 example lines and their panicle detection results using subimages at 7-, 12-, and 20-m flight altitudes are provided in Fig. 4A; also plot-based panicle detection results are included in Fig. S2). The detection accuracy of the Panicle-AI model was evaluated using confusion matrices (Fig. S3) as well as Precision, Recall, F1-score, and mAP@.5 (Table S2).

The correlation analysis was performed between manually counted panicles and the Panicle-AI-derived scoring using subimages collected from the DRPD dataset. For the 7-m flight altitude, a significant correlation (*R*^2^ = 0.945, *P* < 0.001, RMSE = 2.218 panicles per subimage; *n* = 1,524 subimages, representing 41,828 labeled panicles) was obtained, whereas the correlation results based on the 12-m images (*R*^2^ = 0.824, *P* < 0.001, RMSE = 11.596; *n* = 401 subimages, representing 28,889 panicles) and the 20-m images (*R*^2^ = 0.729, *P* < 0.001, RMSE = 22.973; *n* = 224 subimages, representing 32,226 panicles) suggested relatively lower correlations (Fig. 4B). Moreover, correlation analyses on plot-based aerial imagery at GSD_7m_ between AI and manually scored panicles are also provided (Fig. S4). The above analysis indicated that, using the DRPD dataset, the 7-m altitude aerial phenotyping (i.e., GSD = 0.08 to 0.09 cm·pixel^−1^) was suitable for AI-powered detection of rice panicles under field conditions.

**Fig. 5.**
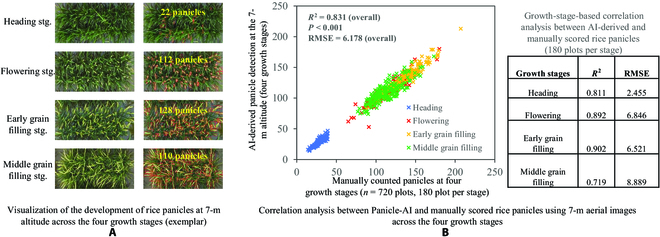
Panicle counting and correlation analysis at 4 growth stages. (A) Visualization of panicle detection at 4 growth stages using plot-based aerial images at GSD_7m_. (B) Correlations between Panicle-AI derived and manually counted rice panicles using 7-m aerial images across the 4 growth stages. Growth stage based correlation analysis of panicle detection is also provided in a table.

After identifying the preferred GSD for panicle detection, we then conducted the correlation analysis between AI-derived and manually scored rice panicles using the 7-m imagery at 4 growth stages. Because of phenotypic variation of panicle numbers at different growth stages (Fig. 5A), we applied the Panicle-AI to analyze 180 plots per stage and concluded an overall correlation (*R*^2^ = 0.831, *P* < 0.001, RMSE = 6.178 panicles per plot; *n* = 480; Fig. 5B, left). Moreover, correlation results at flowering and early grain filling (*R*^2^ = 0.892 and 0.902, RMSE = 6.846 and 6.414) were higher than those at booting (*R*^2^ = 0.811, RMSE = 2.455) and middle grain filling (*R*^2^ = 0.719, RMSE = 8.889), indicating that both flowering and early grain filling are suitable growth stages for panicle phenotyping at the 7-m altitude (Fig. 5B, right).

### The performance of the Panicle-AI model

To verify the performance of Panicle-AI, we first performed ablation experiments using the GSD_7m_ images (Table S3), which determined the necessity of optimized modules in the learning architecture. Then, we compared its panicle detection accuracy against 13 state-of-the-art object detection DL models, including VFNet, CenterNet [52], FCOS, GFLv2, TOOD [53], Cascade R-CNN [54], Faster R-CNN [55], YOLOX [56], YOLOv5, ATSS [57], EfficientNet [58], and RetinaNet, all of which were carefully fine-tuned to yield the best panicle detection using the DRPD dataset. We evaluated the detection results using 7-m (GSD_7m_) aerial images collected at early grain filling (with the best *R*^2^; Fig. 5B), among which the Panicle-AI resulted in the best detection accuracy in terms of mAP@.5 for both flight altitudes (the mAP@.5 values were 0.967). Table presents the 7-m mAP@.5, model parameters, speed [giga floating point operations per second (GFLOPS)], and the model size for all the trained models, with our preferred Panicle-AI model listed on the last row (bolded). The results suggested that the Panicle-AI model performed the best compared with other state-of-the-art AI models on GSD_7m_ images, with 1.3% to 9.2% increases in terms of mAP@.5, much smaller parameters, model size, and faster GFLOPs.

**Table.** Comparisons of all the trained DL models together with the Panicle-AI model using a variety of evaluation metrics.

**Model ID**	**Model**	**mAP@.5**	**Parameters (M)**	**GFLOPs**	**Model size (M)**
1	Faster R-CNN	0.925	41.34	90.8	332.6
2	Cascade R-CNN	0.931	69.152	118.8	557.5
3	RetinaNet	0.941	58.341	128	542.4
4	FCOS	0.926	51.11	111.6	409.5
5	GFLv2	0.944	32.04	81.8	245.5
6	TOOD	0.953	31.79	78.8	257.1
7	VFNet	0.941	32.709	75.5	264.4
8	Dynamic R-CNN	0.875	41.348	90.491	331.8
9	ATSS	0.941	32.113	80.475	259.2
10	CenterNet	0.935	32.1	78.6	257.9
11	EfficientNet	0.948	18.339	78.56	223.4
12	YOLOX	0.951	8.94	26.6	68.5
13	YOLOv5	0.954	6.69	15.8	14.5
**Preferred**	**Panicle-AI**	**0.967**	**8.14**	**28.6**	**17.6**

### The cloud-based platform

**Fig. 6.**
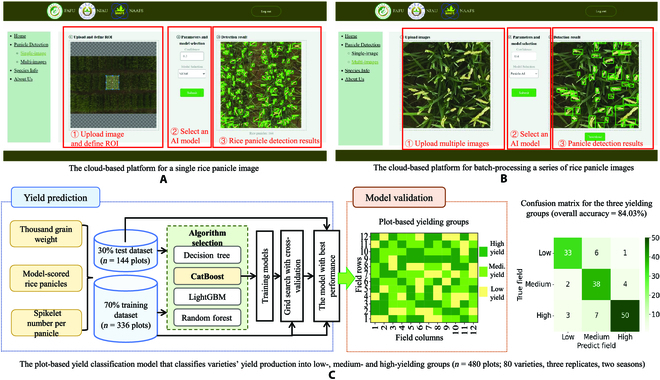
Cloud-based platform for researchers to readily use and to perform yield classification. (A) Cloud-based panicle detection on a single image uploaded by users, including the selection of ROI, choosing a pretrained AI model, and downloading the detection results. (B) Cloud-based batch processing of a series of images. (C) A yield classification model trained with 366 plot-based yield productions and 144 for evaluation.

To facilitate nonexperts to use the Panicle-AI model for panicle detection, we built the Panicle-Cloud platform and integrated 6 AI models that were compatible with the cloud-based runtime environment into the platform, including Panicle-AI, YOLOv5, VFNet, FCOS, GFLv2, and RetinaNet. Users could select a suitable model to detect rice panicles using their own images. The platform used browser–server structure and the MVC design, which was implemented via the AliCloud-based platform together with the database management system for hosting historic datasets and cloud server. After registration, users could use a web browser to log in the platform (http://ai-panicle.com:32123) and then select the single-image page or multi-images webpage from the navigation menu to initiate panicle detection. For the single-image workflow (Fig. 6A), users could (a) upload a single image to the platform, (b) select a region of interest (ROI) to proceed, (c) select a pretrained AI model and define the confidence level, and (d) view the panicle detection result.

Similarly, for the multi-images workflow (Fig. 6B), users could upload a series of images and then batch-process these images with the same settings of ROI, parameters, AI model selection, and result download. To optimize cloud-based computing, we limited the maximum size of a single uploaded image to 30 MB. If the length or width of an uploaded image was greater than 960 pixels, the platform would automatically crop the image into several subimages to achieve an accurate panicle detection and decreased computing complexity. Developers could utilize the back-end system to manage and maintain the platform, including updating the embedded DL models, modifying historic database, downloading detection results, and managing registered user information.

### The yield classification in the 2-season rice field trial

To classify yield groups based on the key yield trait (i.e., PNpM^2^), we chose to combine supervised ML algorithms with the main yield-related factors (Fig. 6C, left) to perform the task. In total, 480 plot-based yield results and plot-level images (80 varieties and 3 replicates) were chosen to train the yield classification model, including PNpM^2^, SNpP, and TGW. Because of limited input parameters, we used 4 supervised ML algorithms (i.e., Decision Trees, CatBoost, LightGBM, and Random Forest; Table S4). On the basis of *k*-fold cross-validation (*k* = 5), we found that the CatBoost model performed the best with the training data (*n* = 336 plot-based yield production) and thus chose it for classifying rice yield production. Then, we adjusted the depth to 5 and random seed to 3 to optimize the model and achieved an accuracy of 83.63% in the cross-validation (*n* = 144 plots; Fig. S5). The weights of PNpM^2^, TGW, and SNpP were set as 0.255, 0.286, and 0.459, respectively. The above was achieved through recommended practices in hyperparameter fine-tuning published previously [59].

Using the CatBoost-based yield classification model, we classified the yielding group into 3 categories (colored yellow, cyan, and green, representing low, medium, and high yield groups, respectively; Fig. 6C, middle). A confusion matrix was produced to demonstrate the performance of the classification based on the testing set (Fig. 6C, right; detailed classification results listed in Table S5): (a) In the low-yielding class, 33 plots (80.5%) were correctly classified, whereas 6 plots (14.6%) were wrongly classified as medium-yielding plots, and only 1 high-yielding plot (2.4%) was classified wrongly; (b) for the medium-yielding class, correct classified plots were 38 (86.4%), and wrongly classified low- and high-yielding plots were 2 (4.5%) and 4 (9.1%), respectively; (c) in the high-yielding class, correctly classified plots were 50 (83.3%), and incorrectly classified low- and medium-yielding plots were 3 (5%) and 7 (11.7%), respectively. Hence, the overall accuracy of the yield classification on the testing set was 84.03%.

## Discussion

Rice is a key crop for many developing countries in Asia and Africa as it provides a variety of nutrients and energy to their rapidly growing population [60]. Breeders, plant researchers, growers, and farmers are confronted with the challenges of improving rice yields to ensure food supply, focusing on improving key yield components in the field. Still, present field phenotyping approaches were limited when characterizing such yield-related traits [26], resulting in the development the Panicle-Cloud platform to automate the measurement of rice panicles from drone-collected aerial images, which quantified the PNpM^2^ trait from over 200 rice varieties cultivated around the world. The platform and the preferred Panicle-AI model were applied to a 2-season rice breeding trial and helped breeders screen and select favorite varieties with an ML-powered yield classification model.

### DRPD—An open dataset for rice panicle detection

Along with the development of vision-based DL and ML techniques, it is evident that open and high-quality training datasets have become a bottleneck for computer vision and plant research communities to fully exploit the potential of AI. The 2022 GWHD dataset [61] contained 275,187 labeled wheat spikes from 6,422 images across 12 countries, which was a successful attempt to supply diverse and DL-ready data annotation to relevant research in wheat. Following this momentum, we established the open DRPD dataset to facilitate rice panicle measurements. The DRPD dataset contained 5,372 RGB images cropped from raw aerial images and 259,498 labeled rice panicles from 229 rice varieties, covering different panicle developmental and morphological features at 4 growth stages and across 12 geo-locations in Asia and North America. More importantly, we identified the preferred GSD for panicle phenotyping (GSD_7m_ = 0.08 to 0.09 cm·pixel^−1^) and, thus, the bigger portion of the labeled rice panicles in the DRPD dataset. Similar to GWHD, we envisage that the DRPD dataset could also be widely used to facilitate the algorithmic development of rice panicle and panicle-like object detection, facilitating yield-based rice phenotyping and related breeding or agronomic activities.

### Drone-based phenotyping to record rice panicle signals

To identify a suitable aerial phenotyping protocol to collect rice panicle images in the field, a high-end camera (45 million pixels) was used to perform field phenotyping at 3 different attitudes (i.e., 7, 12, and 20 m) and across 4 key growth stages (i.e., heading, flowering, early grain filling, and middle grain filling). The acquired images were cropped and used to build the DRPD datasets. From both panicle labeling and object detection’s perspectives, we found that the GSD_7m_ aerial imagery at early grain filling was easy to annotate and has led to high-accuracy panicle detection with a range of trained DL models, indicating that the right timing and image resolution for phenotyping rice panicles. In addition, according to the labeled data in the DRPD, it is noticeable that rice panicles were still under development during heading, making the morphological features of panicles inconsistent; similarly, rice panicles became curved and ripened from middle grain filling onward, generating different structural and color features. Both scenarios could lead to poor rice panicle detection in the field and thus inaccurate analysis of the PNpM^2^ trait.

### The preferred Panicle-AI model and the Panicle-Cloud platform

The Panicle-AI model using YOLOv5 as the baseline learning structure with tailored modules to help the model extract small objects from images even when they were occluded or clustered. For example, the improved PB module was capable of extracting features through enhancing features possessed by small objects, which was further improved by the use of attention mechanism to focus on panicle-like objects. Changing the loss function enabled the model to learn positive samples between dense panicle clusters and thus improved the detection accuracy. In addition, we used the SE block twice in the backbone and once in the neck, which helped enhance the selectivity of semantic information of high-level features together with local information of low-level features. This approach reduced the misdetection due to adjacent rice panicles and noise signals such as leaves, helping the model to detect some occluded rice panicles. More interestingly, using the Panicle-AI model trained with GSD_7m_ aerial images, we could also detect panicles on images with other GSDs (e.g., GSD_12m_ and GSD_20m_), demonstrating the generalization of the model. Nevertheless, we found that when rice panicle signals were very small (e.g., in GSD_20m_ images) or panicles overlapped with each other (e.g., middle grain filling onward), most of the trained AI models were struggling to detect panicles with high accuracy.

As the PNpM^2^ trait is a key indicator for yield potential, we also investigated this trait across 4 growth stages using the Panicle-Cloud platform. We discovered that not only did the PNpM^2^ trait correlate well with the ground truthing at flowering or early grain filling, but the trait acquired at these 2 stages also led to better yield classification results. Still, it is worth noting that, because of the scale of the 2-season field experiments, the model could be continuously improved with training data collected from other rice varieties, which naturally led to the establishment of the Panicle-Cloud platform (http://ai-panicle.com:32123) that could be easily used by nonexperts, integrating AI-powered solutions, labeled rice panicle database (i.e., the DRPD), and rice panicle detection algorithms into an open hub to facilitate the plant research community to cross-validate analysis results and perform yield classification.

### Limitations and future developments

The Panicle-Cloud platform presented here combined several DL models, computer vision algorithms, and cloud computing techniques into the detection of a key yield-related trait, PNpM^2^, in rice. Because of its modular design, plant researchers and developers could extend the platform relatively easily. In addition, the DRPD dataset was a key first step toward achieving a more comprehensive data-driven foundation for AI-powered panicle detection, which could be used to train more powerful and generalized AI models. Still, it is clear that more plant researchers and breeders’ involvement will help the Panicle-Cloud platform and the DRPD dataset achieve a major step change in future yield-based phenotypic analysis work.

In addition, more historic rice yield production data will enable the platform to further improve the yield classification model, which was mainly trained with representative rice varieties cultivated in mainland China. Together with the development of both yield- and image-based datasets collected from field phenotyping, key yield-related traits collected at different growth stages could also be utilized to improve the classification accuracy by incorporating developmental patterns into yield classification.

In addition, we trust that the Panicle-Cloud platform will provide users with the ability to accommodate and compare AI models using panicle detection results. Because of its open-source nature, DL techniques such as quantization, model pruning, and knowledge distillation should be applied to the cloud computing, enhancing the throughput and capability of model training and verification by different research groups around the world. The platform could also consider using images collected by other phenotyping approaches such as handheld devices and gantry systems, which would capture more detailed panicle-level features compared to those collected by drones. Through this approach, a multiscale panicle phenotyping method is likely to be established to enable the quantification of other yield-related traits to facilitate more reproducible yield-related detection and prediction.

## Conclusions

Rice (*O. sativa*) is an essential stable food for many rice consumption nations in the world. To evaluate rice varieties’ yield performance under complex field conditions, key yield-related traits such as PNpM^2^ provide good in-field indicators, which are challenging to quantify at a large scale due to phenotypic variation of rice cultivars and panicle-level morphological features. Here, we present the Panicle-Cloud platform, an open and AI-powered cloud computing platform that is capable of quantifying rice panicles from drone-collected imagery using a range of AI models. To build the model, we first established an open DRPD dataset consisting of 229 rice varieties with 259,498 panicles annotated by a group of rice specialists. Then, we modified the learning architecture of a YOLO-based DL model and developed the Panicle-AI model, which was integrated into the Panicle-Cloud platform, so that nonexpert users could use and detect rice panicles from their own aerial images. After trialing the platform in a 2-season trial, we validated the biological relevance of our work, leading the establishment of a yield classification model using the AI-derived PNpM^2^ trait. Hence, we trust that our work presents valuable advances in AI- and cloud-powered phenotyping under field conditions, providing rice breeders and plant researchers a useful toolkit to perform yield-related trait analysis to screen and select desired varieties in rice.

## Acknowledgments

We would like to thank all members of the Digital Fujian Research Institute of Big Data for Agriculture and Forestry, Fujian Agriculture and Forestry University (FAFU, China), as well as members from the UK-China Zhou laboratory. In particular, we would like to thank Z. Lan, Z. Lin, F. Zhuang, and A. Swanepoel for technical supports in training dataset. **Funding:** This work was partially supported by the National Natural Science Foundation of China (under grant nos. 32070400, 62171130, 61972093, and 61802064), in part by the Fujian University Industry University Research Joint Innovation Project under grant 2022H6006, and in part by the Fujian Science and Technology Planning Project under grant 2021S0007. Drone-based phenotypic analysis and yield prediction were supported by the National Natural Science Foundation of China (32 070 400 to J.Z.). Both J.Z. and R.J. were partially supported by the United Kingdom Research and Innovation's (UKRI) Biotechnology and Biological Sciences Research Council’s (BBSRC) International Partnership Grant (BB/X511882/1). **Author contributions:** J.Z. and C.Y. wrote the manuscript with inputs from Z.T. and J.C. J.C., S.W., and Z.T. designed the Panicle-Cloud platform under J.Z. and C.Y.’s supervision, with help from J.W. and R.C. J.W. performed the rice experiments and UAV-based field phenotyping. J.W., R.J., and L.S. provided expertise in aerial imaging and crop modeling. Y.L. and J.C. tested and optimized the software. J.Z., C.Y., and Z.T. performed the data analysis. Z.T., L.S., and C.Y. performed yield analysis under J.Z.’s supervision. All authors read and approved the final manuscript. **Competing interests:** The authors declare that they have no competing interests.

## Data Availability

Release page and source code can be found via https://github.com/changcaiyang/Panicle-AI/releases/; the DRPD dataset: 5,372 RGB subimages with annotate 259,498 panicles collected from 229 rice varieties can also be downloaded for the GitHub repository. The cloud-based platform can be accessed via http://ai-panicle.com:32123, which requires a user to register a user account first. A test account is provided: username: user1 and password: 110110.

## Supplementary Materials

Supplementary 1 Fig. S1. Changes in evaluation metrics and loss functions during the training of the Panicle-AI model on the 7-m dataset.
Fig. S2. Visualization of three different GSDs for different rice plot imagery (exemplar).
Fig. S3. The results of confusion matrices in aerial images acquired at the different altitudes (i.e. GSD_7m_, GSD_12m_, and GSD_20m_, respectively).
Fig. S4. Correlation analysis between the Panicle-AI scored and manually scored rice panicles based on 7-m aerial images together with correlation analysis based on in-field panicle scoring.
Fig. S5. Actual yield distribution and model-predicted yielding groups of 144 experimental plots.
Table S1. Plot number in the testing set (within a cell: upper, trial year; lower, variety ID) for yield classification.
Table S2. Result of panicle detection using aerial images collected at three altitudes (i.e., 3 different GSDs).
Table S3. Ablation experiments with the Panicle-AI model on the 7-m test set.
Table S4. Yield classification results of several machine learning models using the test set of yield production.
Table S5. The yield production test set for the yield classification modeling.
Movie S1. GUI of the Panicle-Cloud platform in operation.
