# Supplementary material for: Panicle-Cloud: An Open and AI-Powered Cloud Computing Platform for Quantifying Rice Panicles from Drone-Collected Imagery to Enable the Classification of Yield Production in Rice
Source: Plant Phenomics. 2023 Oct 16;5:0105. doi: 10.34133/plantphenomics.0105 (PMC10578299; doi:10.34133/plantphenomics.0105)
Supplement: Supplementary 1 — Fig. S1. Changes in evaluation metrics and loss functions during the training of the Panicle-AI model on the 7-m dataset. Fig. S2. Visualization of three different GSDs for different rice plot imagery (exemplar). Fig. S3. The results of confusion matrices in aerial images acquired at the different altitudes (i.e. GSD7m, GSD12m, and GSD20m, respectively). Fig. S4. Correlation analysis between the Panicle-AI scored and manually scored rice panicles based on 7-m aerial images together with correlation analysis based on in-field panicle scoring. Fig. S5. Actual yield distribution and model-predicted yielding groups of 144 experimental plots. Table S1. Plot number in the testing set (within a cell: upper, trial year; lower, variety ID) for yield classification. Table S2. Result of panicle detection using aerial images collected at three altitudes (i.e., 3 different GSDs). Table S3. Ablation experiments with the Panicle-AI model on the 7-m test set. Table S4. Yield classification results of several machine learning models using the test set of yield production. Table S5. The yield production test set for the yield classification modeling. Movie S1. GUI of the Panicle-Cloud platform in operation. [file plantphenomics.0105.f1.zip › PlantPhenomics-D-23-00102_Supplementary materials.docx]

Supporting Information

**Figure S1. Changes in evaluation metrics and loss functions during the training of the Panicle-AI model on the 7-m dataset.**


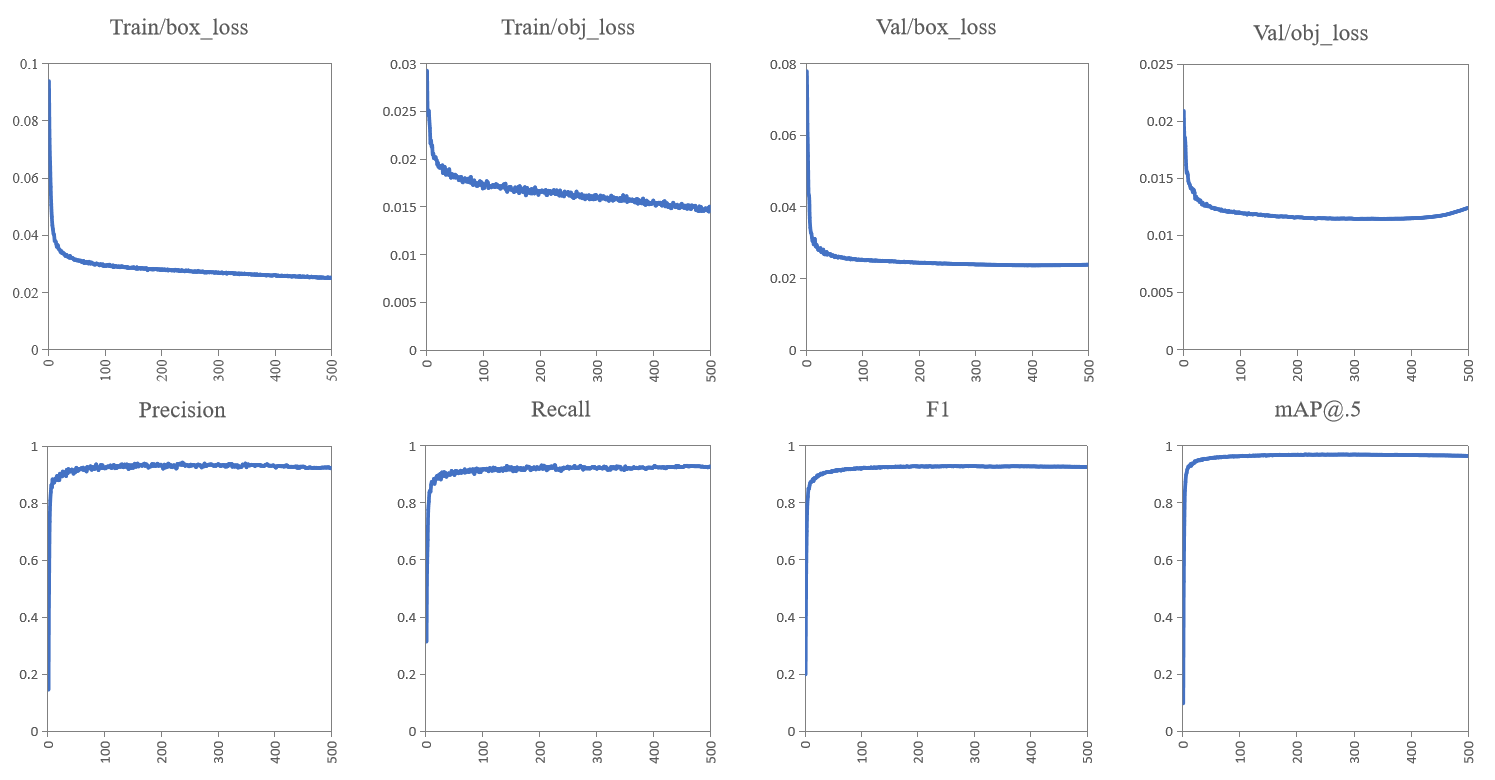


**Figure S2. Visualization of three different GSDs for different rice plot imagery (exemplar).**


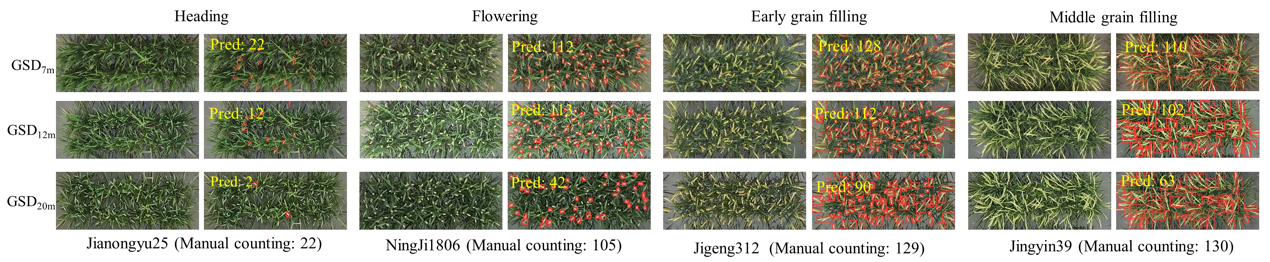


**Figure S3. The results of Confusion matrices in aerial images acquired at the different altitudes (i.e. GSD_7m_, GSD_12m,_ and GSD_20m_, respectively).**


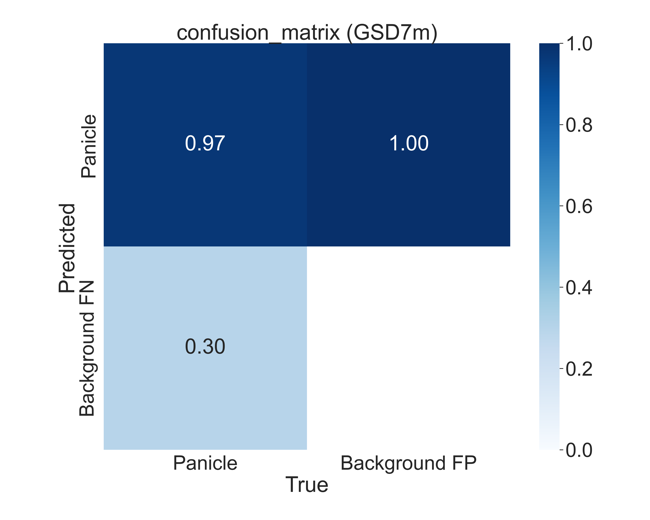

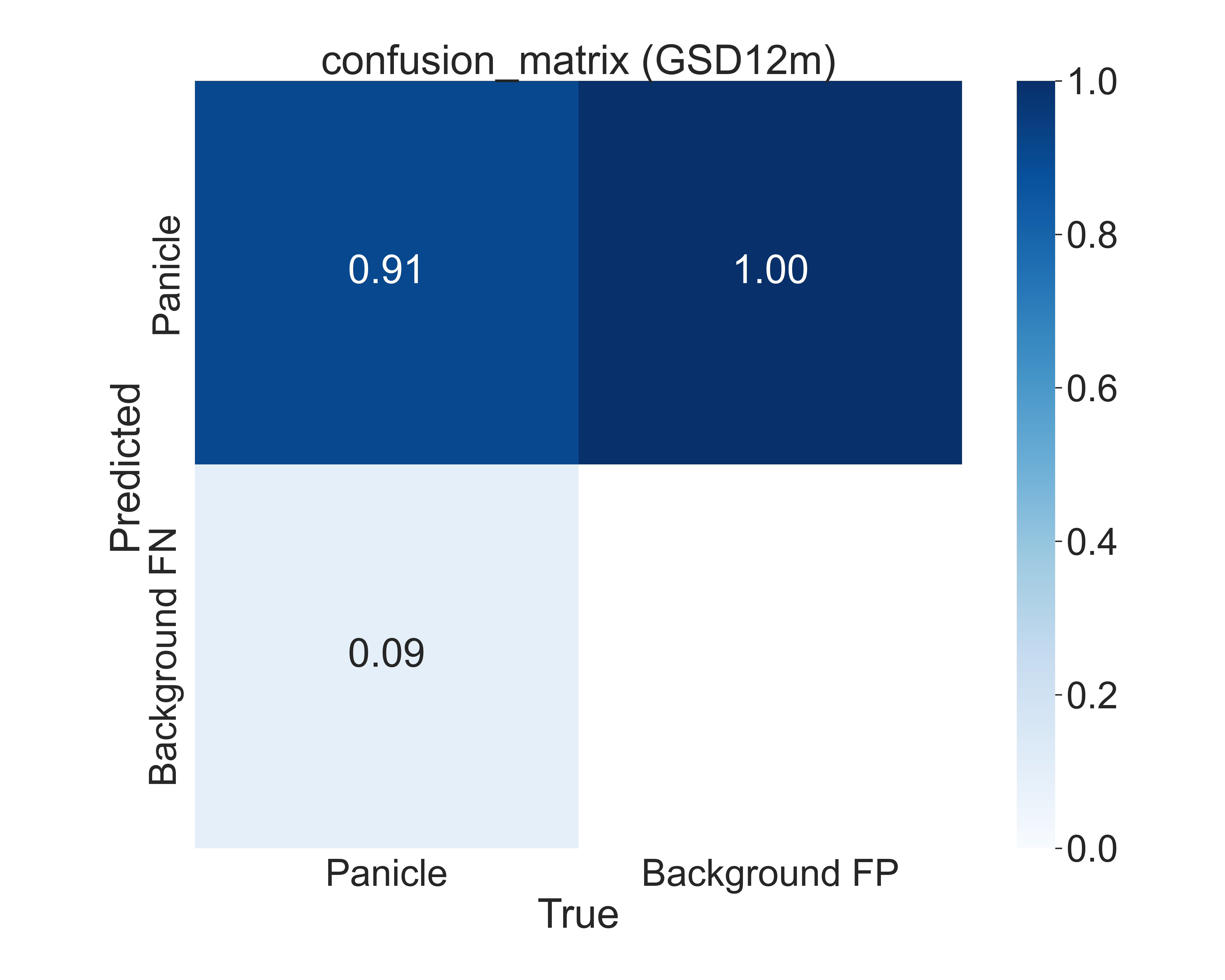


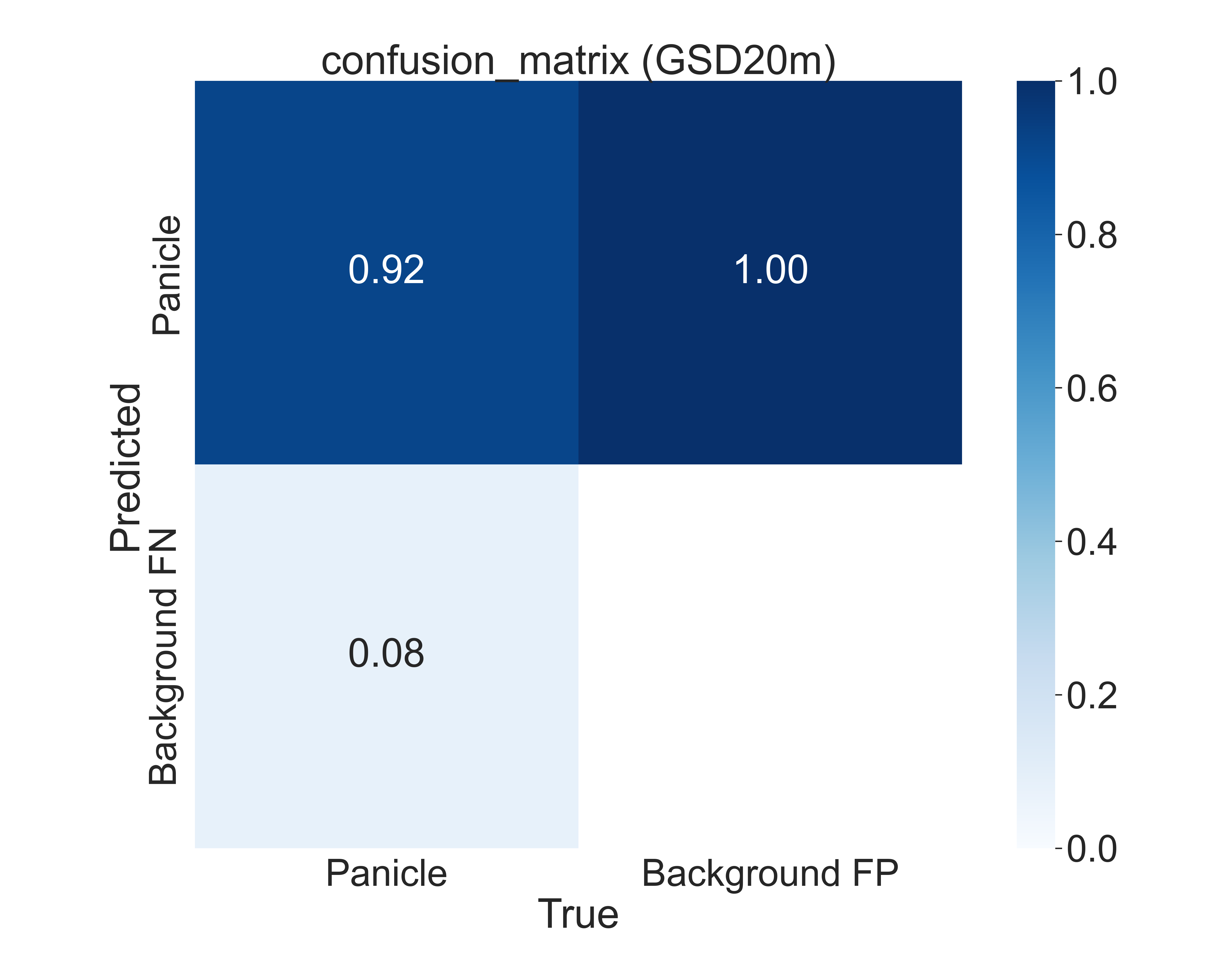


**Figure S4. Correlation analysis between the Panicle-AI scored and manually scored rice panicles based on 7-m aerial images together with correlation analysis based on in-field panicle scoring.**

**
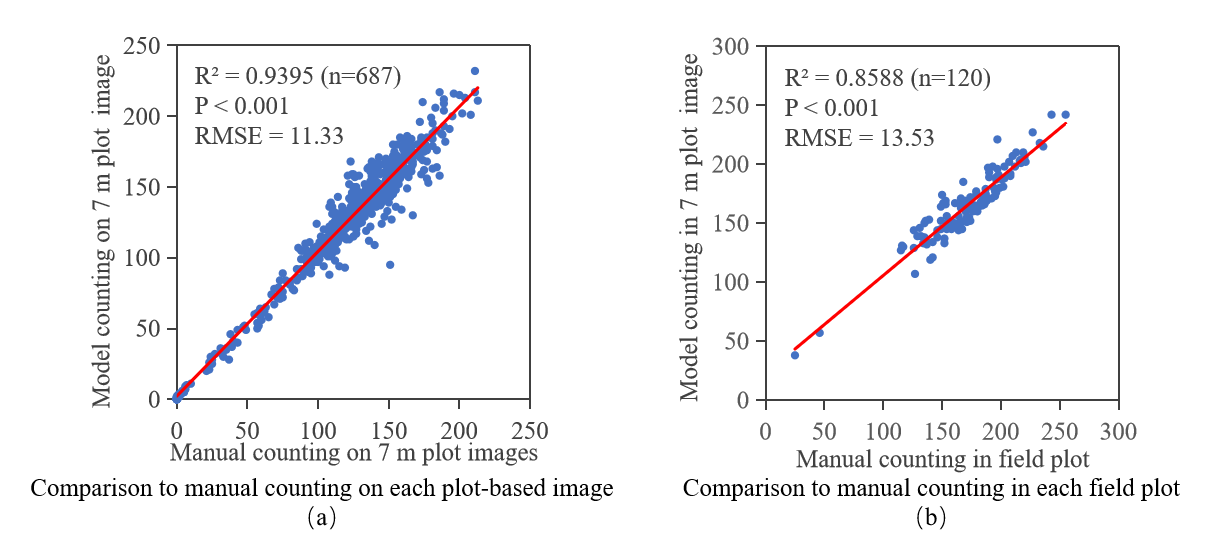
**

**Figure S5. Actual yield distribution and model-predicted yielding groups of 144 experimental plots.**


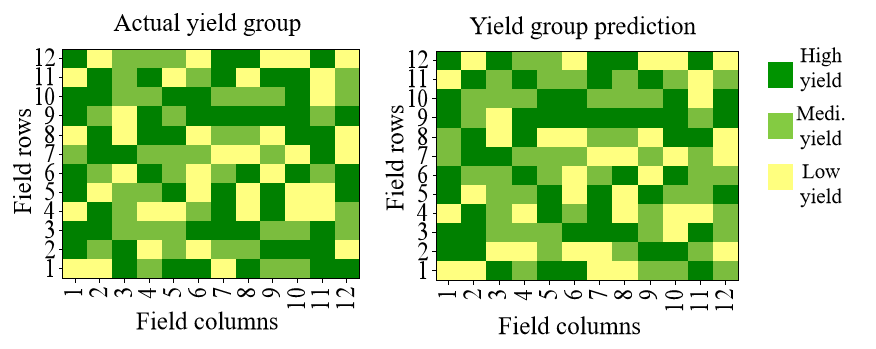


**Table S1. Plot number in the testing set (within a cell: upper, trial year; lower, variety ID) for yield classification.**

| 21N3039 | 21N2082 | 22N3169 | 21N1007 | 22N1018 | 22N3086 | 21N2042 | 22N2139 | 21N1034 | 21N1072 | 21N1156 | 21N2066 |
| --- | --- | --- | --- | --- | --- | --- | --- | --- | --- | --- | --- |
| 21N3143 | 21N2161 | 22N2166 | 22N1144 | 22N1023 | 21N3050 | 22N3042 | 21N2075 | 22N1163 | 21N2047 | 21N3035 | 21N3082 |
| 22N2029 | 22N3035 | 21N3150 | 21N1172 | 22N2078 | 22N1038 | 22N1051 | 21N1015 | 21N1083 | 22N1139 | 21N2139 | 22N1172 |
| 22N1077 | 22N3038 | 21N2156 | 22N2039 | 21N1029 | 22N3081 | 21N2173 | 22N3052 | 22N2018 | 22N1052 | 21N2079 | 21N2052 |
| 21N3014 | 22N3015 | 21N2078 | 22N2086 | 22N1070 | 21N3169 | 21N1170 | 22N1169 | 22N3069 | 22N2085 | 22N3172 | 21N3023 |
| 22N3006 | 22N1075 | 22N3030 | 22N1046 | 22N2072 | 22N2023 | 21N1176 | 21N2072 | 22N1176 | 21N2014 | 22N1026 | 21N1169 |
| 22N1153 | 21N1036 | 21N3075 | 22N3150 | 21N1006 | 22N2014 | 22N2143 | 22N1024 | 21N3066 | 22N3047 | 22N3154 | 21N1025 |
| 21N2033 | 21N3020 | 22N3018 | 22N1047 | 22N3075 | 21N3011 | 22N1006 | 21N3154 | 21N3083 | 21N3168 | 21N1010 | 21N1024 |
| 21N3164 | 21N1031 | 22N1035 | 22N1015 | 21N1173 | 22N3062 | 22N2052 | 21N3076 | 22N2062 | 21N1014 | 21N3019 | 21N2163 |
| 22N2050 | 22N3024 | 21N1022 | 21N3062 | 22N1049 | 21N1076 | 22N1011 | 22N2169 | 22N1012 | 22N2034 | 21N1163 | 21N1033 |
| 21N2039 | 22N3085 | 22N3037 | 21N1084 | 21N2085 | 21N2069 | 21N1047 | 21N1085 | 22N1084 | 22N3161 | 21N2035 | 22N2056 |
| 21N1086 | 22N1027 | 21N2174 | 21N2028 | 21N1144 | 22N3143 | 22N2154 | 22N3070 | 21N3041 | 22N1157 | 22N3040 | 21N2043 |

**Table S2. Result of panicle detection using aerial images collected at three altitudes (i.e. three different GSDs).**

| GSD | Task | Precision | Recall | F1 | [mAP@.5](mailto:mAP@.5) |
| --- | --- | --- | --- | --- | --- |
| GSD_7m_ | val | 0.934 | 0.924 | 0.929 | 0.968 |
|  | test | 0.921 | 0.928 | 0.924 | 0.967 |
| GSD_12m_ | val | 0.8468 | 0.8394 | 0.8431 | 0.8823 |
|  | test | 0.8395 | 0.841 | 0.8402 | 0.8786 |
| GSD_20m_ | val | 0.8815 | 0.8447 | 0.8627 | 0.9124 |
|  | test | 0.8816 | 0.8294 | 0.8547 | 0.9051 |

**Table S3. Ablation experiments with the Panicle-AI model on the 7-m test set.**

|  | Precision | Recall | F1 | mAP@.5 |
| --- | --- | --- | --- | --- |
| YOLOv5s | 0.926 | 0.916 | 0.921 | 0.954 |
| Panicle-C3 | 0.927 | 0.920 | 0.923 | 0.958 |
| Panicle-C3+SE | 0.924 | 0.923 | 0.923 | 0.963 |
| Panicle-C3+SE+VFL | 0.921 | 0.928 | 0.924 | 0.967 |

**Table S4. Yield classification results of several machine learning models using the test set of yield production.**

|  | **Class** | **Precision** | **Recall** | **F1-score** | **Accuracy** |
| --- | --- | --- | --- | --- | --- |
| **Finetune_CatBoost** | Low | 0.82 | 0.87 | 0.85 | 0.8403 |
|  | Medium | 0.83 | 0.91 | 0.87 |  |
|  | High | 0.86 | 0.75 | 0.80 |  |
| **CatBoost** | Low | 0.82 | 0.85 | 0.84 | 0.8194 |
|  | Medium | 0.84 | 0.71 | 0.77 |  |
|  | High | 0.80 | 0.91 | 0.85 |  |
| **Decision Tree** | Low | 0.85 | 0.83 | 0.84 | 0.8056 |
|  | Medium | 0.82 | 0.71 | 0.76 |  |
|  | High | 0.77 | 0.88 | 0.82 |  |
| **LightGBM** | Low | 0.82 | 0.82 | 0.82 | 0.7917 |
|  | Medium | 0.75 | 0.70 | 0.73 |  |
|  | High | 0.80 | 0.84 | 0.82 |  |
| **Random Forest** | Low | 0.80 | 0.86 | 0.83 | 0.8125 |

**Table S5. The yield-production test set for the yield classification modelling.**

| Number | Thousand-grain weight | The number of spikelets per panicle | Panicles | Class |
| --- | --- | --- | --- | --- |
| 2021N1086 | 25.5 | 121.6764 | 111 | low |
| 2021N2039 | 24.6 | 92 | 168 | high |
| 2022N2050 | 25.8 | 95.2 | 145 | high |
| 2021N3164 | 23 | 107.358 | 97 | low |
| 2021N2033 | 25 | 106 | 146 | high |
| 2022N1153 | 24.5 | 78 | 146 | high |
| 2022N3006 | 29 | 55 | 140 | Medium |
| 2021N3014 | 27 | 60 | 133 | low |
| 2022N1077 | 23.8 | 127.309 | 152 | high |
| 2022N2029 | 26.5 | 90 | 123 | high |
| 2022N3085 | 23.6 | 117.5265 | 209 | Medium |
| 2021N3039 | 24.6 | 92 | 145 | high |
| 2022N1027 | 23.5 | 69.1635 | 91 | low |
| 2021N3143 | 25.2 | 106.812 | 102 | low |
| 2022N3024 | 27 | 92 | 143 | high |
| 2021N1031 | 26 | 110 | 148 | high |
| 2021N3020 | 27 | 90 | 89 | low |
| 2021N1036 | 22.5 | 90 | 143 | Medium |
| 2022N1075 | 26.8 | 102.6186 | 149 | high |
| 2022N3015 | 28 | 70 | 147 | high |
| 2022N3038 | 24 | 85 | 115 | Medium |
| 2022N3035 | 25 | 87.5 | 132 | high |
| 2021N2161 | 21.3 | 121.2224 | 151 | high |
| 2021N2174 | 25.3 | 98.2795 | 175 | high |
| 2021N2082 | 23.8 | 128.3513 | 111 | low |
| 2022N3037 | 24.5 | 78 | 110 | high |
| 2021N1022 | 22.6 | 50.5 | 145 | Medium |
| 2022N1035 | 25 | 87.5 | 129 | Medium |
| 2022N3018 | 25.8 | 81 | 124 | Medium |
| 2021N3075 | 26.8 | 102.6186 | 132 | low |
| 2022N3030 | 26 | 75 | 138 | high |
| 2022N2062 | 24.2 | 72.4 | 126 | high |
| 2021N3083 | 22.3 | 129.0465 | 156 | high |
| 2021N3150 | 24.9 | 106.6956 | 126 | Medium |
| 2022N2166 | 25 | 60 | 142 | Medium |
| 2022N3169 | 25.7 | 109.12 | 141 | Medium |
| 2021N2028 | 27.8 | 77 | 132 | Medium |
| 2021N1084 | 23.1 | 110.89 | 106 | low |
| 2021N3062 | 24.2 | 72.4 | 123 | Medium |
| 2022N1015 | 28 | 70 | 123 | low |
| 2022N1047 | 25.5 | 99 | 125 | Medium |
| 2022N3150 | 24.9 | 106.6956 | 150 | high |
| 2022N1046 | 24.7 | 96 | 130 | Medium |
| 2022N2086 | 25.5 | 121.6764 | 171 | high |
| 2022N2039 | 24.6 | 92 | 139 | high |
| 2021N1172 | 23 | 99.408 | 150 | Medium |
| 2022N1144 | 24.2 | 72.4 | 152 | high |
| 2021N1007 | 26.5 | 64 | 155 | Medium |
| 2021N1144 | 24.2 | 72.4 | 146 | high |
| 2022N3086 | 25.5 | 121.6764 | 116 | low |
| 2022N1049 | 25 | 90 | 133 | Medium |
| 2021N1173 | 21.2 | 128.275 | 147 | low |
| 2022N3075 | 26.8 | 102.6186 | 149 | high |
| 2021N1006 | 29 | 55 | 144 | Medium |
| 2022N2072 | 28.739 | 77.28 | 129 | Medium |
| 2022N1070 | 24.8 | 109.7 | 121 | high |
| 2021N1029 | 26.5 | 90 | 132 | Medium |
| 2022N2078 | 24.1 | 119.3872 | 160 | high |
| 2022N1023 | 22 | 86 | 120 | low |
| 2022N1018 | 25.8 | 81 | 136 | Medium |
| 2022N3143 | 25.2 | 106.812 | 141 | high |
| 2021N2069 | 26.3 | 86 | 135 | low |
| 2021N1076 | 24.5 | 115.4 | 148 | high |
| 2022N3062 | 24.2 | 72.4 | 123 | Medium |
| 2021N3011 | 26 | 80 | 100 | low |
| 2022N2014 | 27 | 60 | 127 | low |
| 2022N2023 | 22 | 86 | 134 | Medium |
| 2021N3169 | 25.7 | 109.12 | 108 | low |
| 2022N3081 | 24 | 125.6879 | 155 | high |
| 2022N1038 | 24 | 85 | 159 | high |
| 2021N3050 | 25.8 | 95.2 | 114 | Medium |
| 2021N2085 | 23.6 | 117.5265 | 145 | Medium |
| 2022N2154 | 25 | 90 | 113 | low |
| 2021N1047 | 25.5 | 99 | 96 | Medium |
| 2022N1011 | 26 | 80 | 150 | high |
| 2022N2052 | 23.9 | 101.8 | 185 | high |
| 2022N1006 | 29 | 55 | 175 | high |
| 2022N2143 | 25.2 | 106.812 | 127 | Medium |
| 2021N1176 | 25 | 101.6595 | 113 | low |
| 2021N1170 | 23.8 | 128.3513 | 143 | Medium |
| 2021N2173 | 21.2 | 128.275 | 160 | high |
| 2022N1051 | 25.7 | 89.8 | 130 | Medium |
| 2022N3042 | 26.6 | 80.91 | 144 | high |
| 2021N2042 | 26.6 | 80.91 | 142 | high |
| 2022N3070 | 24.8 | 109.7 | 119 | high |
| 2021N1085 | 23.6 | 117.5265 | 141 | Medium |
| 2022N2169 | 25.7 | 109.12 | 142 | high |
| 2021N3076 | 24.5 | 115.4 | 122 | low |
| 2021N3154 | 25 | 90 | 110 | low |
| 2022N1024 | 27 | 92 | 140 | high |
| 2021N2072 | 28.739 | 77.28 | 113 | low |
| 2022N1169 | 25.7 | 109.12 | 133 | Medium |
| 2022N3052 | 23.9 | 101.8 | 147 | high |
| 2021N1015 | 28 | 70 | 133 | Medium |
| 2021N2075 | 26.8 | 102.6186 | 115 | low |
| 2022N2139 | 23.5 | 111.104 | 169 | high |
| 2021N3041 | 24.5 | 74 | 125 | Medium |
| 2022N1084 | 23.1 | 110.89 | 155 | high |
| 2022N1012 | 28 | 50 | 139 | Medium |
| 2021N2078 | 24.1 | 119.3872 | 113 | low |
| 2021N2156 | 28.9 | 112.9625 | 123 | low |
| 2021N3066 | 26.9 | 91.8 | 122 | low |
| 2022N1176 | 25 | 101.6595 | 148 | Medium |
| 2022N3069 | 26.3 | 86 | 60 | low |
| 2022N2018 | 25.8 | 81 | 145 | high |
| 2021N1083 | 22.3 | 129.0465 | 140 | Medium |
| 2022N1163 | 22.4 | 119.9589 | 172 | high |
| 2021N1034 | 25 | 90 | 97 | low |
| 2022N1157 | 24.8 | 121.1565 | 168 | Medium |
| 2022N3161 | 21.3 | 121.2224 | 154 | high |
| 2022N2034 | 25 | 90 | 118 | Medium |
| 2021N1014 | 27 | 60 | 124 | low |
| 2021N3168 | 25.6 | 95.8226 | 102 | low |
| 2022N3047 | 25.5 | 99 | 139 | high |
| 2021N2014 | 27 | 60 | 122 | low |
| 2022N2085 | 23.6 | 117.5265 | 178 | high |
| 2022N1052 | 23.9 | 101.8 | 166 | high |
| 2022N1139 | 23.5 | 111.104 | 154 | high |
| 2021N2047 | 25.5 | 99 | 129 | high |
| 2021N1072 | 28.739 | 77.28 | 110 | low |
| 2022N3040 | 22.4 | 79.97 | 145 | high |
| 2021N2035 | 25 | 87.5 | 132 | high |
| 2021N1163 | 22.4 | 119.9589 | 162 | high |
| 2021N3019 | 26 | 100 | 98 | low |
| 2021N1010 | 25.5 | 121.5852 | 125 | low |
| 2022N3154 | 25 | 90 | 127 | Medium |
| 2022N1026 | 23.8 | 74.7 | 121 | high |
| 2022N3172 | 23 | 99.408 | 181 | high |
| 2021N2079 | 22.2 | 123.5162 | 140 | Medium |
| 2021N2139 | 23.5 | 111.104 | 111 | low |
| 2021N3035 | 25 | 87.5 | 98 | low |
| 2021N1156 | 28.9 | 112.9625 | 157 | high |
| 2021N2043 | 23.5 | 98 | 153 | high |
| 2022N2056 | 23 | 84 | 111 | low |
| 2021N1033 | 25 | 106 | 116 | Medium |
| 2021N2163 | 22.4 | 119.9589 | 138 | Medium |
| 2021N1024 | 27 | 92 | 141 | high |
| 2021N1025 | 25 | 70 | 134 | high |
| 2021N1169 | 25.7 | 109.12 | 114 | low |
| 2021N3023 | 22 | 86 | 114 | low |
| 2021N2052 | 23.9 | 101.8 | 164 | high |
| 2022N1172 | 23 | 99.408 | 161 | Medium |
| 2021N3082 | 23.8 | 128.3513 | 132 | Medium |
| 2021N2066 | 26.9 | 91.8 | 126 | low |

**Movies S1. GUI of the Panicle-Cloud platform in operation.**
